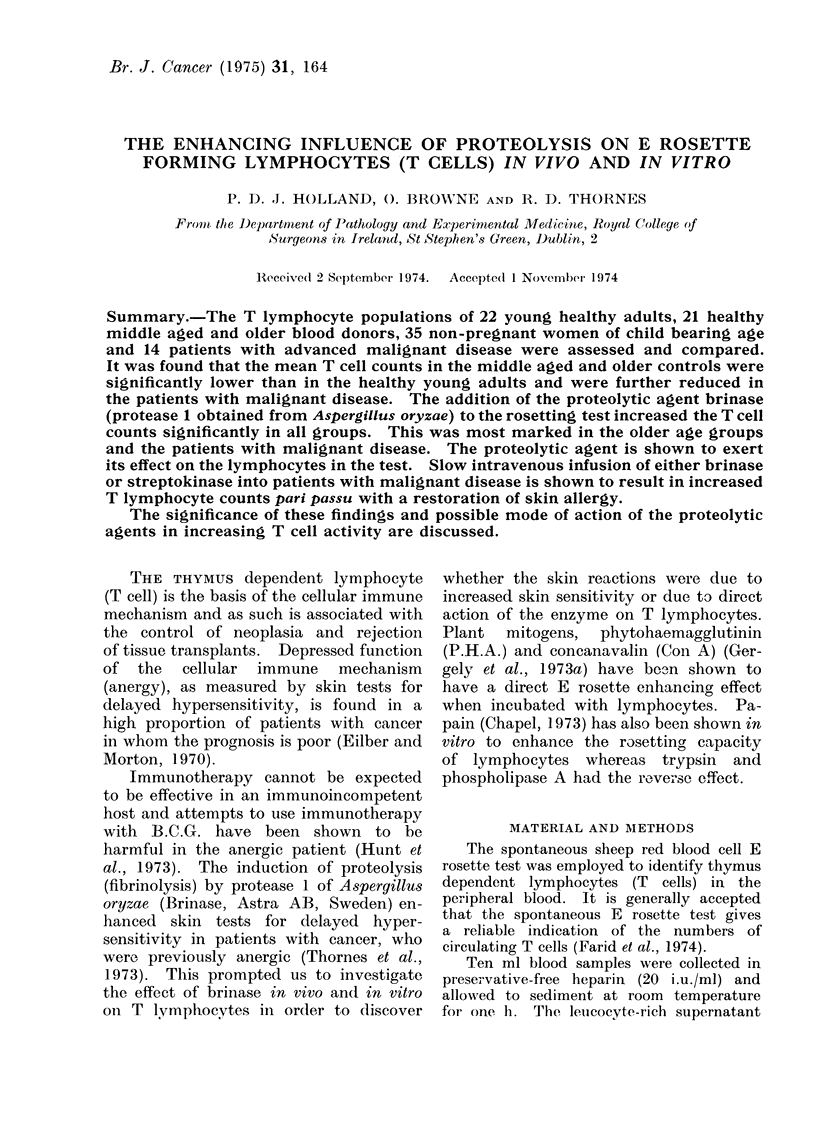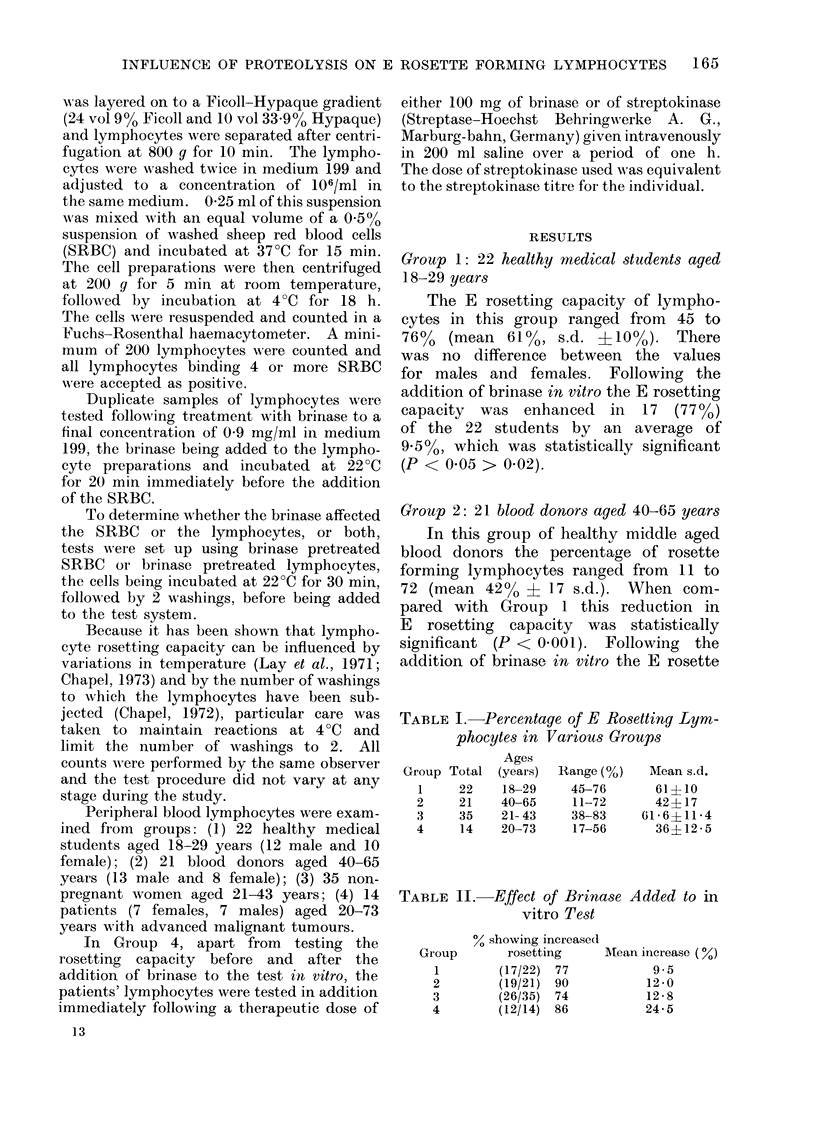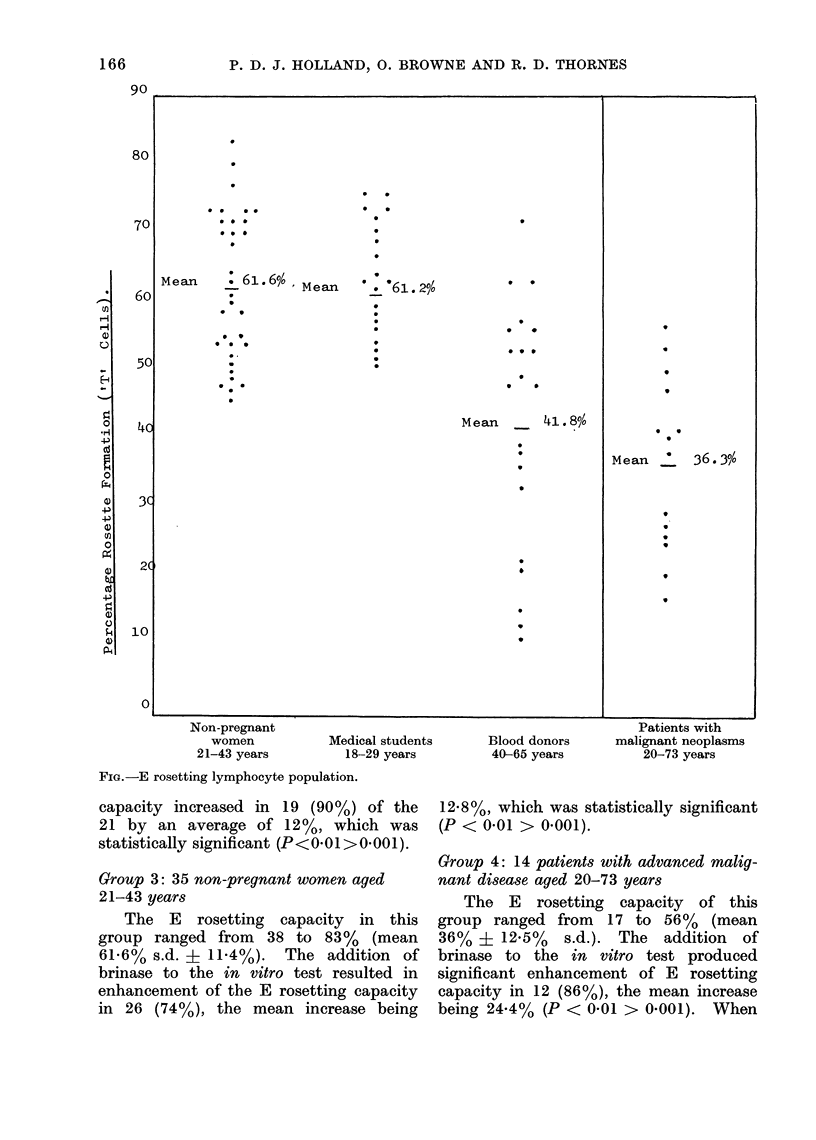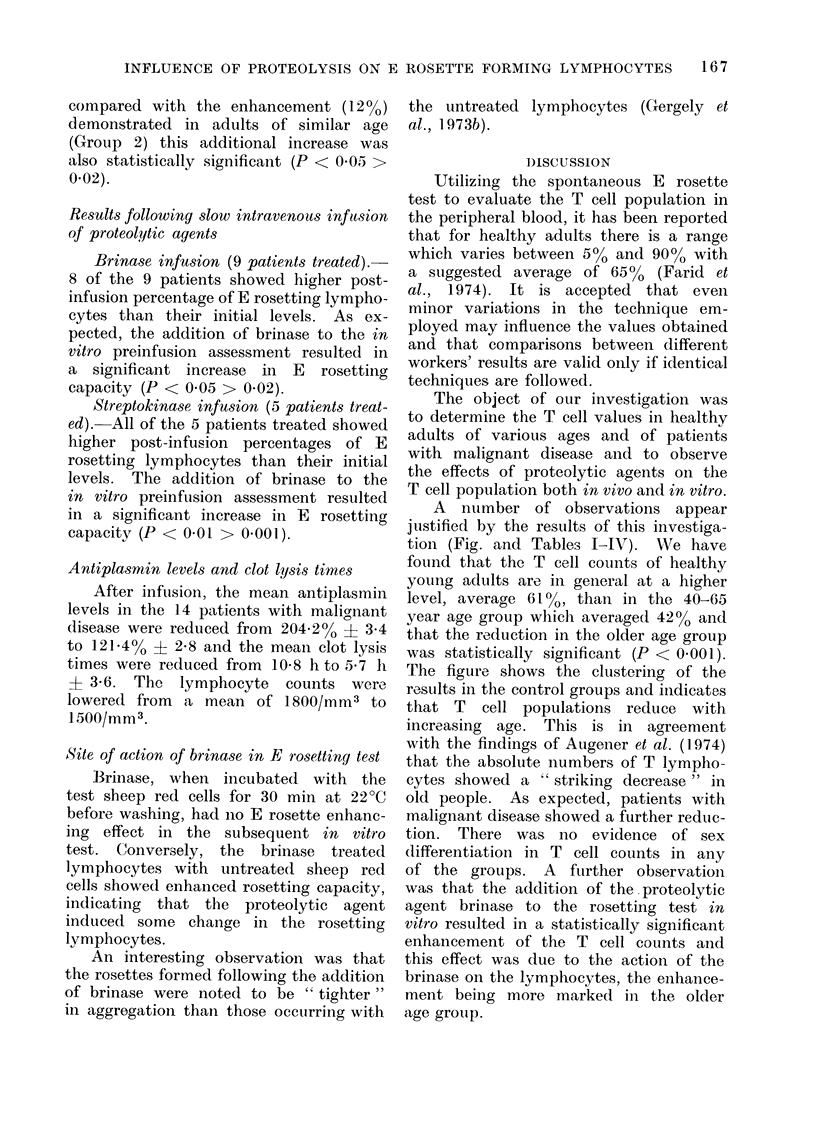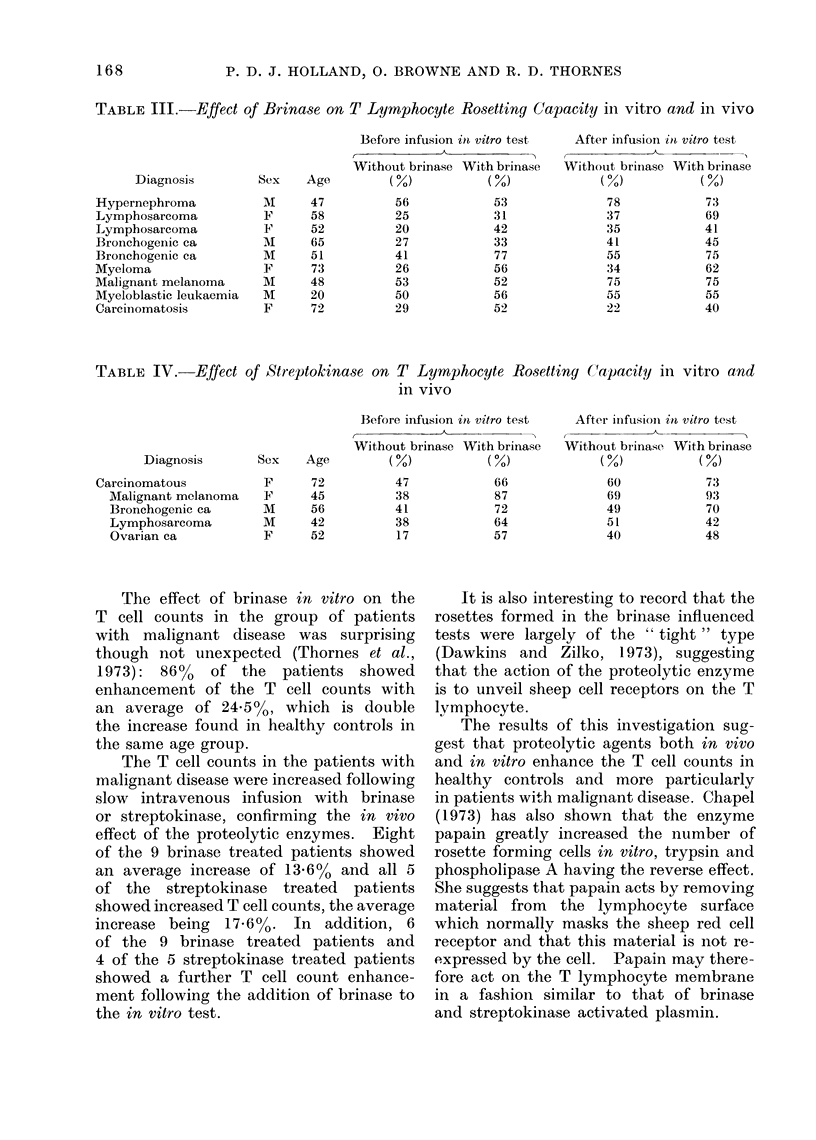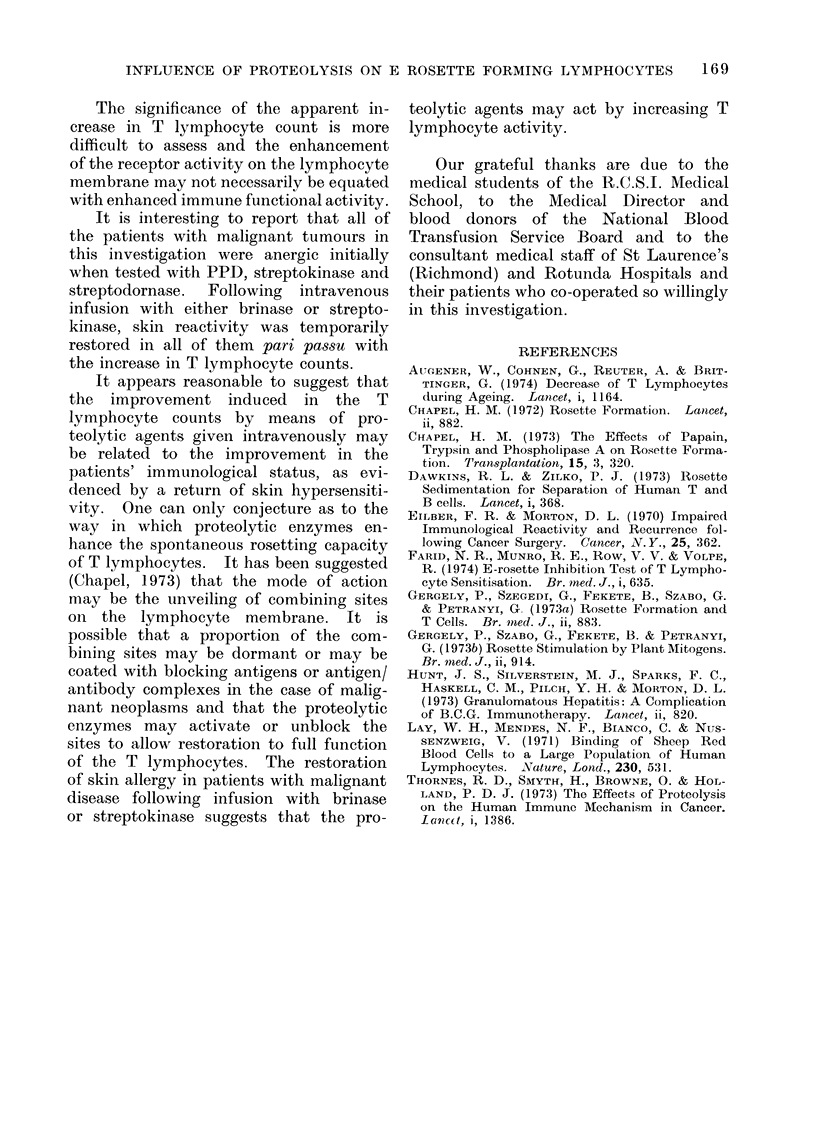# The enhancing influence of proteolysis on E rosette forming lymphocytes (T cells) in vivo and in vitro.

**DOI:** 10.1038/bjc.1975.21

**Published:** 1975-02

**Authors:** P. D. Holland, O. Browne, R. D. Thornes

## Abstract

The T lymphocytes populations of 22 young healthy adults, 21 healthy middle aged and older blood donors, 35 non-pregnant women of child bearing age and 14 patients with advanced malignant disease were assessed and compared. It was found that the mean T cell counts in the middle aged and older controls were significantly lower than in the healthy young adults and were further reduced in the patients with malignant disease. The addition of the proteolytic agent brinase (protease 1 obtained from Aspergillus oryzae) to the rosetting test increased the T cell counts signficantly in all groups. This was mot marked in the older age groups and the patients with malignant disease. The proteolytic agent is shown to exert its effect on the lymphocytes in the test. Slow intravenous infusion of either brinase or streptokinase into patients with malignant disease is shown to result in increased T lymphocyte counts pari passu with a restoration of skin allergy. The significance of these findings and possible mode of action of the proteolytic agents in increasing T cell activity are discussed.


Br. J. Cancer (1975) 31, 164

### THE ENHANCING INFLUENCE OF PROTEOLYSIS ON E ROSETTE FORMING LYMPHOCYTES (T CELLS) *IN VIVO* AND *IN VITRO*

P. D. J. HOLLAND, O. BROWNE AND R. D. THORNES

*From the Department of Pathology and Experimental Medicine, Royal College of Surgeons in Ireland, St Stephen's Green, Dublin, 2*

Received 2 September 1974. Accepted 1 November 1974

**Summary.—The T lymphocyte populations of 22 young healthy adults, 21 healthy middle aged and older blood donors, 35 non-pregnant women of child bearing age and 14 patients with advanced malignant disease were assessed and compared. It was found that the mean T cell counts in the middle aged and older controls were significantly lower than in the healthy young adults and were further reduced in the patients with malignant disease. The addition of the proteolytic agent brinase (protease 1 obtained from *Aspergillus oryzae*) to the rosetting test increased the T cell counts significantly in all groups. This was most marked in the older age groups and the patients with malignant disease. The proteolytic agent is shown to exert its effect on the lymphocytes in the test. Slow intravenous infusion of either brinase or streptokinase into patients with malignant disease is shown to result in increased T lymphocyte counts *pari passu* with a restoration of skin allergy.**

**The significance of these findings and possible mode of action of the proteolytic agents in increasing T cell activity are discussed.**

THE THYMUS dependent lymphocyte (T cell) is the basis of the cellular immune mechanism and as such is associated with the control of neoplasia and rejection of tissue transplants. Depressed function of the cellular immune mechanism (anergy), as measured by skin tests for delayed hypersensitivity, is found in a high proportion of patients with cancer in whom the prognosis is poor (Eilber and Morton, 1970).

Immunotherapy cannot be expected to be effective in an immunoincompetent host and attempts to use immunotherapy with B.C.G. have been shown to be harmful in the anergic patient (Hunt *et al.*, 1973). The induction of proteolysis (fibrinolysis) by protease 1 of *Aspergillus oryzae* (Brinase, Astra AB, Sweden) enhanced skin tests for delayed hypersensitivity in patients with cancer, who were previously anergic (Thornes *et al.*, 1973). This prompted us to investigate the effect of brinase *in vivo* and *in vitro* on T lymphocytes in order to discover whether the skin reactions were due to increased skin sensitivity or due to direct action of the enzyme on T lymphocytes. Plant mitogens, phytohaemagglutinin (P.H.A.) and concanavalin (Con A) (Gergely *et al.*, 1973*a*) have been shown to have a direct E rosette enhancing effect when incubated with lymphocytes. Papain (Chapel, 1973) has also been shown *in vitro* to enhance the rosetting capacity of lymphocytes whereas trypsin and phospholipase A had the reverse effect.

### MATERIAL AND METHODS

The spontaneous sheep red blood cell E rosette test was employed to identify thymus dependent lymphocytes (T cells) in the peripheral blood. It is generally accepted that the spontaneous E rosette test gives a reliable indication of the numbers of circulating T cells (Farid *et al.*, 1974).

Ten ml blood samples were collected in preservative-free heparin (20 i.u./ml) and allowed to sediment at room temperature for one h. The leucocyte-rich supernatant was layered on to a Ficoll–Hypaque gradient (24 vol 9% Ficoll and 10 vol 33·9% Hypaque) and lymphocytes were separated after centrifugation at 800 *g* for 10 min. The lymphocytes were washed twice in medium 199 and adjusted to a concentration of $10^6$/ml in the same medium. 0·25 ml of this suspension was mixed with an equal volume of a 0·5% suspension of washed sheep red blood cells (SRBC) and incubated at 37°C for 15 min. The cell preparations were then centrifuged at 200 *g* for 5 min at room temperature, followed by incubation at 4°C for 18 h. The cells were resuspended and counted in a Fuchs–Rosenthal haemacytometer. A minimum of 200 lymphocytes were counted and all lymphocytes binding 4 or more SRBC were accepted as positive.

Duplicate samples of lymphocytes were tested following treatment with brinase to a final concentration of 0·9 mg/ml in medium 199, the brinase being added to the lymphocyte preparations and incubated at 22°C for 20 min immediately before the addition of the SRBC.

To determine whether the brinase affected the SRBC or the lymphocytes, or both, tests were set up using brinase pretreated SRBC or brinase pretreated lymphocytes, the cells being incubated at 22°C for 30 min, followed by 2 washings, before being added to the test system.

Because it has been shown that lymphocyte rosetting capacity can be influenced by variations in temperature (Lay *et al.*, 1971; Chapel, 1973) and by the number of washings to which the lymphocytes have been subjected (Chapel, 1972), particular care was taken to maintain reactions at 4°C and limit the number of washings to 2. All counts were performed by the same observer and the test procedure did not vary at any stage during the study.

Peripheral blood lymphocytes were examined from groups: (1) 22 healthy medical students aged 18–29 years (12 male and 10 female); (2) 21 blood donors aged 40–65 years (13 male and 8 female); (3) 35 non-pregnant women aged 21–43 years; (4) 14 patients (7 females, 7 males) aged 20–73 years with advanced malignant tumours.

In Group 4, apart from testing the rosetting capacity before and after the addition of brinase to the test *in vitro*, the patients' lymphocytes were tested in addition immediately following a therapeutic dose of either 100 mg of brinase or of streptokinase (Streptase-Hoechst Behringwerke A. G., Marburg-bahn, Germany) given intravenously in 200 ml saline over a period of one h. The dose of streptokinase used was equivalent to the streptokinase titre for the individual.

### RESULTS

*Group* 1: 22 *healthy medical students aged* 18–29 *years*

The E rosetting capacity of lymphocytes in this group ranged from 45 to 76% (mean 61%, s.d. ±10%). There was no difference between the values for males and females. Following the addition of brinase *in vitro* the E rosetting capacity was enhanced in 17 (77%) of the 22 students by an average of 9·5%, which was statistically significant ($P < 0{\cdot}05 > 0{\cdot}02$).

*Group* 2: 21 *blood donors aged* 40–65 *years*

In this group of healthy middle aged blood donors the percentage of rosette forming lymphocytes ranged from 11 to 72 (mean 42% ± 17 s.d.). When compared with Group 1 this reduction in E rosetting capacity was statistically significant ($P < 0{\cdot}001$). Following the addition of brinase *in vitro* the E rosette

TABLE I.—*Percentage of E Rosetting Lymphocytes in Various Groups*

| Group | Total | Ages (years) | Range (%) | Mean s.d. |
|---|---|---|---|---|
| 1 | 22 | 18–29 | 45–76 | 61±10 |
| 2 | 21 | 40–65 | 11–72 | 42±17 |
| 3 | 35 | 21-43 | 38–83 | 61·6±11·4 |
| 4 | 14 | 20–73 | 17–56 | 36±12·5 |

TABLE II.—*Effect of Brinase Added to* in vitro *Test*

| Group | % showing increased rosetting | | Mean increase (%) |
|---|---|---|---|
| 1 | (17/22) | 77 | 9·5 |
| 2 | (19/21) | 90 | 12·0 |
| 3 | (26/35) | 74 | 12·8 |
| 4 | (12/14) | 86 | 24·5 |

FIG.—E rosetting lymphocyte population.

capacity increased in 19 (90%) of the 21 by an average of 12%, which was statistically significant ($P<0{\cdot}01>0{\cdot}001$).

*Group* 3: 35 *non-pregnant women aged* 21–43 *years*

The E rosetting capacity in this group ranged from 38 to 83% (mean 61·6% s.d. ± 11·4%). The addition of brinase to the *in vitro* test resulted in enhancement of the E rosetting capacity in 26 (74%), the mean increase being 12·8%, which was statistically significant ($P < 0{\cdot}01 > 0{\cdot}001$).

*Group* 4: 14 *patients with advanced malignant disease aged* 20–73 *years*

The E rosetting capacity of this group ranged from 17 to 56% (mean 36% ± 12·5% s.d.). The addition of brinase to the *in vitro* test produced significant enhancement of E rosetting capacity in 12 (86%), the mean increase being 24·4% ($P < 0{\cdot}01 > 0{\cdot}001$). When compared with the enhancement (12%) demonstrated in adults of similar age (Group 2) this additional increase was also statistically significant ($P < 0{\cdot}05 > 0{\cdot}02$).

*Results following slow intravenous infusion of proteolytic agents*

*Brinase infusion* (*9 patients treated*).—8 of the 9 patients showed higher post-infusion percentage of E rosetting lymphocytes than their initial levels. As expected, the addition of brinase to the *in vitro* preinfusion assessment resulted in a significant increase in E rosetting capacity ($P < 0{\cdot}05 > 0{\cdot}02$).

*Streptokinase infusion* (*5 patients treated*).—All of the 5 patients treated showed higher post-infusion percentages of E rosetting lymphocytes than their initial levels. The addition of brinase to the *in vitro* preinfusion assessment resulted in a significant increase in E rosetting capacity ($P < 0{\cdot}01 > 0{\cdot}001$).

*Antiplasmin levels and clot lysis times*

After infusion, the mean antiplasmin levels in the 14 patients with malignant disease were reduced from 204·2% ± 3·4 to 121·4% ± 2·8 and the mean clot lysis times were reduced from 10·8 h to 5·7 h ± 3·6. The lymphocyte counts were lowered from a mean of 1800/mm$^3$ to 1500/mm$^3$.

*Site of action of brinase in E rosetting test*

Brinase, when incubated with the test sheep red cells for 30 min at 22°C before washing, had no E rosette enhancing effect in the subsequent *in vitro* test. Conversely, the brinase treated lymphocytes with untreated sheep red cells showed enhanced rosetting capacity, indicating that the proteolytic agent induced some change in the rosetting lymphocytes.

An interesting observation was that the rosettes formed following the addition of brinase were noted to be " tighter " in aggregation than those occurring with the untreated lymphocytes (Gergely *et al.*, 1973*b*).

DISCUSSION

Utilizing the spontaneous E rosette test to evaluate the T cell population in the peripheral blood, it has been reported that for healthy adults there is a range which varies between 5% and 90% with a suggested average of 65% (Farid *et al.*, 1974). It is accepted that even minor variations in the technique employed may influence the values obtained and that comparisons between different workers' results are valid only if identical techniques are followed.

The object of our investigation was to determine the T cell values in healthy adults of various ages and of patients with malignant disease and to observe the effects of proteolytic agents on the T cell population both *in vivo* and *in vitro*.

A number of observations appear justified by the results of this investigation (Fig. and Tables I–IV). We have found that the T cell counts of healthy young adults are in general at a higher level, average 61%, than in the 40–65 year age group which averaged 42% and that the reduction in the older age group was statistically significant ($P < 0{\cdot}001$). The figure shows the clustering of the results in the control groups and indicates that T cell populations reduce with increasing age. This is in agreement with the findings of Augener *et al.* (1974) that the absolute numbers of T lymphocytes showed a " striking decrease " in old people. As expected, patients with malignant disease showed a further reduction. There was no evidence of sex differentiation in T cell counts in any of the groups. A further observation was that the addition of the proteolytic agent brinase to the rosetting test *in vitro* resulted in a statistically significant enhancement of the T cell counts and this effect was due to the action of the brinase on the lymphocytes, the enhancement being more marked in the older age group.

TABLE III.—*Effect of Brinase on T Lymphocyte Rosetting Capacity* in vitro *and* in vivo

| | | | Before infusion *in vitro* test | | After infusion *in vitro* test | |
|---|---|---|---|---|---|---|
| Diagnosis | Sex | Age | Without brinase (%) | With brinase (%) | Without brinase (%) | With brinase (%) |
| Hypernephroma | M | 47 | 56 | 53 | 78 | 73 |
| Lymphosarcoma | F | 58 | 25 | 31 | 37 | 69 |
| Lymphosarcoma | F | 52 | 20 | 42 | 35 | 41 |
| Bronchogenic ca | M | 65 | 27 | 33 | 41 | 45 |
| Bronchogenic ca | M | 51 | 41 | 77 | 55 | 75 |
| Myeloma | F | 73 | 26 | 56 | 34 | 62 |
| Malignant melanoma | M | 48 | 53 | 52 | 75 | 75 |
| Myeloblastic leukaemia | M | 20 | 50 | 56 | 55 | 55 |
| Carcinomatosis | F | 72 | 29 | 52 | 22 | 40 |

TABLE IV.—*Effect of Streptokinase on T Lymphocyte Rosetting Capacity* in vitro *and* in vivo

| | | | Before infusion *in vitro* test | | After infusion *in vitro* test | |
|---|---|---|---|---|---|---|
| Diagnosis | Sex | Age | Without brinase (%) | With brinase (%) | Without brinase (%) | With brinase (%) |
| Carcinomatous | F | 72 | 47 | 66 | 60 | 73 |
| Malignant melanoma | F | 45 | 38 | 87 | 69 | 93 |
| Bronchogenic ca | M | 56 | 41 | 72 | 49 | 70 |
| Lymphosarcoma | M | 42 | 38 | 64 | 51 | 42 |
| Ovarian ca | F | 52 | 17 | 57 | 40 | 48 |

The effect of brinase *in vitro* on the T cell counts in the group of patients with malignant disease was surprising though not unexpected (Thornes *et al.*, 1973): 86% of the patients showed enhancement of the T cell counts with an average of 24·5%, which is double the increase found in healthy controls in the same age group.

The T cell counts in the patients with malignant disease were increased following slow intravenous infusion with brinase or streptokinase, confirming the *in vivo* effect of the proteolytic enzymes. Eight of the 9 brinase treated patients showed an average increase of 13·6% and all 5 of the streptokinase treated patients showed increased T cell counts, the average increase being 17·6%. In addition, 6 of the 9 brinase treated patients and 4 of the 5 streptokinase treated patients showed a further T cell count enhancement following the addition of brinase to the *in vitro* test.

It is also interesting to record that the rosettes formed in the brinase influenced tests were largely of the "tight" type (Dawkins and Zilko, 1973), suggesting that the action of the proteolytic enzyme is to unveil sheep cell receptors on the T lymphocyte.

The results of this investigation suggest that proteolytic agents both *in vivo* and *in vitro* enhance the T cell counts in healthy controls and more particularly in patients with malignant disease. Chapel (1973) has also shown that the enzyme papain greatly increased the number of rosette forming cells *in vitro*, trypsin and phospholipase A having the reverse effect. She suggests that papain acts by removing material from the lymphocyte surface which normally masks the sheep red cell receptor and that this material is not re-expressed by the cell. Papain may therefore act on the T lymphocyte membrane in a fashion similar to that of brinase and streptokinase activated plasmin.

The significance of the apparent increase in T lymphocyte count is more difficult to assess and the enhancement of the receptor activity on the lymphocyte membrane may not necessarily be equated with enhanced immune functional activity.

It is interesting to report that all of the patients with malignant tumours in this investigation were anergic initially when tested with PPD, streptokinase and streptodornase. Following intravenous infusion with either brinase or streptokinase, skin reactivity was temporarily restored in all of them *pari passu* with the increase in T lymphocyte counts.

It appears reasonable to suggest that the improvement induced in the T lymphocyte counts by means of proteolytic agents given intravenously may be related to the improvement in the patients' immunological status, as evidenced by a return of skin hypersensitivity. One can only conjecture as to the way in which proteolytic enzymes enhance the spontaneous rosetting capacity of T lymphocytes. It has been suggested (Chapel, 1973) that the mode of action may be the unveiling of combining sites on the lymphocyte membrane. It is possible that a proportion of the combining sites may be dormant or may be coated with blocking antigens or antigen/antibody complexes in the case of malignant neoplasms and that the proteolytic enzymes may activate or unblock the sites to allow restoration to full function of the T lymphocytes. The restoration of skin allergy in patients with malignant disease following infusion with brinase or streptokinase suggests that the proteolytic agents may act by increasing T lymphocyte activity.

Our grateful thanks are due to the medical students of the R.C.S.I. Medical School, to the Medical Director and blood donors of the National Blood Transfusion Service Board and to the consultant medical staff of St Laurence's (Richmond) and Rotunda Hospitals and their patients who co-operated so willingly in this investigation.

### REFERENCES

AUGENER, W., COHNEN, G., REUTER, A. & BRITTINGER, G. (1974) Decrease of T Lymphocytes during Ageing. *Lancet*, i, 1164.

CHAPEL, H. M. (1972) Rosette Formation. *Lancet*, ii, 882.

CHAPEL, H. M. (1973) The Effects of Papain, Trypsin and Phospholipase A on Rosette Formation. *Transplantation*, **15**, 3, 320.

DAWKINS, R. L. & ZILKO, P. J. (1973) Rosette Sedimentation for Separation of Human T and B cells. *Lancet*, i, 368.

EILBER, F. R. & MORTON, D. L. (1970) Impaired Immunological Reactivity and Recurrence following Cancer Surgery. *Cancer, N.Y.*, **25**, 362.

FARID, N. R., MUNRO, R. E., ROW, V. V. & VOLPE, R. (1974) E-rosette Inhibition Test of T Lymphocyte Sensitisation. *Br. med. J.*, i, 635.

GERGELY, P., SZEGEDI, G., FEKETE, B., SZABO, G. & PETRANYI, G. (1973*a*) Rosette Formation and T Cells. *Br. med. J.*, ii, 883.

GERGELY, P., SZABO, G., FEKETE, B. & PETRANYI, G. (1973*b*) Rosette Stimulation by Plant Mitogens. *Br. med. J.*, ii, 914.

HUNT, J. S., SILVERSTEIN, M. J., SPARKS, F. C., HASKELL, C. M., PILCH, Y. H. & MORTON, D. L. (1973) Granulomatous Hepatitis: A Complication of B.C.G. Immunotherapy. *Lancet*, ii, 820.

LAY, W. H., MENDES, N. F., BIANCO, C. & NUSSENZWEIG, V. (1971) Binding of Sheep Red Blood Cells to a Large Population of Human Lymphocytes. *Nature, Lond.*, **230**, 531.

THORNES, R. D., SMYTH, H., BROWNE, O. & HOLLAND, P. D. J. (1973) The Effects of Proteolysis on the Human Immune Mechanism in Cancer. *Lancet*, i, 1386.